# Multidrug-Resistant *Acinetobacter baumannii* Infections in the United Kingdom versus Egypt: Trends and Potential Natural Products Solutions

**DOI:** 10.3390/antibiotics12010077

**Published:** 2023-01-01

**Authors:** Wafaa H. Elwakil, Soha S. Rizk, Ali M. El-Halawany, Mostafa E. Rateb, Ahmed S. Attia

**Affiliations:** 1 Microbiology and Immunology Postgraduate Program, Faculty of Pharmacy, Cairo University, Cairo 11562, Egypt; 2 Department of Pharmacognosy, Faculty of Pharmacy, Cairo University, Cairo 11562, Egypt; 3 School of Computing, Engineering & Physical Sciences, University of the West of Scotland, Paisley PA1 2BE, UK; 4 Department of Microbiology and Immunology, Faculty of Pharmacy, Cairo University, Cairo 11562, Egypt

**Keywords:** *A. baumannii*, United Kingdom, Egypt, carbapenem resistance, natural products

## Abstract

*Acinetobacter baumannii* is a problematic pathogen of global concern. It causes multiple types of infection, especially among immunocompromised individuals in intensive care units. One of the most serious concerns related to this pathogen is its ability to become resistant to almost all the available antibiotics used in clinical practice. Moreover, it has a great tendency to spread this resistance at a very high rate, crossing borders and affecting healthcare settings across multiple economic levels. In this review, we trace back the reported incidences in the PubMed and the Web of Science databases of *A. baumannii* infections in both the United Kingdom and Egypt as two representative examples for countries of two different economic levels: high and low–middle income countries. Additionally, we compare the efforts made by researchers from both countries to find solutions to the lack of available treatments by looking into natural products reservoirs. A total of 113 studies reporting infection incidence were included, with most of them being conducted in Egypt, especially the recent ones. On the one hand, this pathogen was detected in the UK many years before it was reported in Egypt; on the other hand, the contribution of Egyptian researchers to identifying a solution using natural products is more notable than that of researchers in the UK. Tracing the prevalence of *A. baumannii* infections over the years showed that the infections are on the rise, especially in Egypt vs. the UK. Further concerns are linked to the spread of antibiotic resistance among the isolates collected from Egypt reaching very alarming levels. Studies conducted in the UK showed earlier inclusion of high-throughput technologies in the tracking and detection of *A. baumannii* and its resistance than those conducted in Egypt. Possible explanations for these variations are analyzed and discussed.

## 1. Introduction

*Acinetobacter baumannii* has been regarded for a long time as a harmless microbe, but over the past three decades, it has started to be recognized as a serious pathogen, especially, in the hospital environment [1]. Currently, *A. baumannii* is a nightmare for healthcare communities worldwide due to its notorious ability to develop and acquire resistance to almost all the drugs available in our antibiotic arsenal [2]. The genetic makeup of *A. baumannii* allows it to deploy a diverse array of mechanisms to become antibiotic resistant, including the production of β-lactamases and other degrading enzymes, gene expression regulation, and mutations [3]. Special attention is directed towards carbapenem-resistant *A. baumannii* (CRAB), which has been ranked by the World Health Organization (WHO) as the first in the list of pathogens for which new treatment development is urgently needed [4].

*A. baumannii* can cause multiple types of infections; however, the most serious ones are bacteremia and ventilator-associated pneumonia (VAP). This threat is highly aggravated among patients in intensive care units (ICUs), where the mortality rates can rise to more than 40% [3]. Reports of outbreaks involving *A. baumannii* are coming from all over the world, yet it was noted that the reports published from low-income countries (LIC) may be low in number than those in high-income ones. This was attributed to lack of resources for pathogen detection and identification and even publishing results rather than low prevalence of *A. baumannii* in these settings [5].

The pathogenesis of *A. baumannii* is attributed to several virulence factors that have been identified over time [3]. They include physical factors, such as its surface hydrophobicity; outer membrane structures, such as the outer membrane protein A (OmpA) [6]; and lipopolysaccharides (LPS) and sophisticated secretory machineries, such as Type II [7] and Type VI [8] secretion systems, as well as their substrates [9,10]. In addition to these, this pathogen also has essential nutrients acquisition systems [11,12]. Collectively, these virulence factors and others help *A. baumannii* to form biofilms, colonize the host, evade being killed by host defenses, and establish infection in various organs of the human body [3,13]. 

The infections rates of *A. baumannii* vary worldwide; for example, in the USA, it was estimated in 2019 to be 8500 infections per year with a mortality rate ~8% [14]. On the other side of the Atlantic Ocean, in Europe, the infection rates ranged from 2 to 8%, with most of the infections originating in eastern and southern Europe rather than the countries in the northern and western regions [15]. In Africa, despite the scarcity of data, there is strong evidence that *A. baumannii*, including CRAB, is spreading among the population of this continent [16]. Similar trends were seen in Southeast Asia, where 10.7–23.3% of the hospital-acquired infections (HAIs) were linked to *A. baumannii* [17]. Moreover, in Latin America and the Caribbean region, CRAB represents an emerging health problem [18].

An emerging approach to face the *A. baumannii* antibiotic resistance crises is to seek alternative strategies in order to identify effective sustainable treatment options [19]. Natural products (NPs) represent a good repertoire for potential active moieties that can be effective antimicrobial agents. NPs have always been known as a valuable source for the discovery of antibacterial agents. Several studies have reported the effectiveness of NPs and their isolated compounds as antibacterial agents against Gram-negative resistant bacteria, including *A. baumannii* [20]. Moreover, great advancements in the technologies employed to separate, purify, and identify biologically active ingredients have allowed for a more comprehensive understanding of these compounds [20].

In the current review, we present a detailed overview and comparison of *A. baumannii* infections that were reported in both the United Kingdom (UK) and Egypt as examples of high-income countries (HIC) and low–middle income countries (LMIC), respectively. In addition, we present and discuss the contributions of researchers from both countries in finding NPs that could help to solve the antibiotic resistance problem associated with this formidable pathogen.

## 2. Methodology

The PubMed database (https://pubmed.ncbi.nlm.nih.gov/, accessed on 31 October 2022), updated on 31st of October 2022, was searched using the terms [(UK [Affiliation] OR United Kingdom [Affiliation]) AND (baumannii)] and [(Egypt) AND (baumannii)]. The retrieved results were sorted according to publication date in ascending order. No other limits were applied on the search criteria. Furthermore, the Web of Science (WOS) database (https://www.webofscience.com/, accessed on 16 December 2022) was searched using the following terms: i. UK (All Fields) and baumannii (All Fields) refined by Country/Region: ENGLAND or SCOTLAND or WALES or NORTHERN IREAND, and ii. Egypt (All Fields) and baumannii (All Fields) refined by Country/Region: EGYPT.

**Figure 1:**
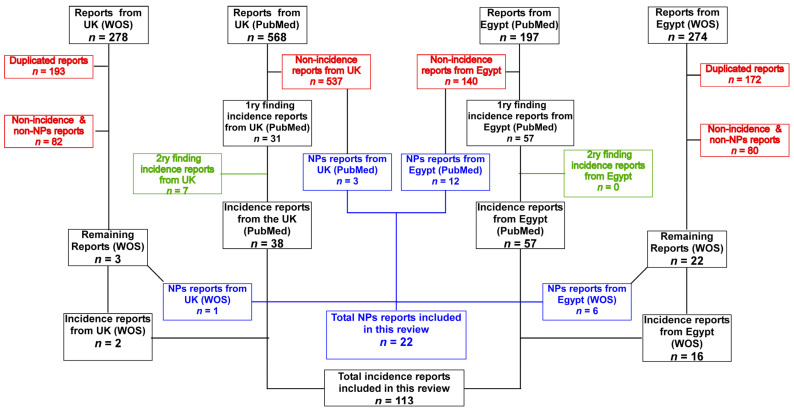
Flow diagram of the search strategy and selection of articles adopted in the current review. The 1ry findings were detected from the main database search, while the 2ry ones are those that were found upon examining the 1ry findings, and the information was cited from the 2ry after their retrieval and examination.

A total of 653 and 299 hits were obtained for the UK and Egypt, respectively, after removing the repeated hits in the two searched databases. The retrieved records were analyzed by two independent researchers applying the following criteria: Studies on *A. baumannii* isolates involved in human infection, either hospital- or community-acquired, that occurred in either the UK or Egypt were included. Furthermore, studies including authors with affiliations connected to either the UK or Egypt but not dealing with isolates obtained and/or analyzed in the respective countries were excluded. Studies mainly concerned with basic or molecular research on standard strains were excluded. However, the studies that included work by British or Egyptian researchers testing natural products against *A. baumannii*, in this case studies including standard strains and/or clinical isolates, were included. Finally, repeated studies that appeared in both searches for the UK and Egypt were only kept and counted once. A summary of the search strategy applied in the current study is presented in Figure 1.

A total of 106 main studies were included, with 7 secondary studies being identified after inspection of the primary ones retrieved from the original search. A total of 22 studies regarded testing NPs. Special notes on the identification of *A. baumannii* strains, their antibiotic susceptibility patterns, and whether they were either multidrug-resistant (MDR) or CRAB were recorded. In addition, special notes were recorded about the molecular characterization that was performed on the isolates that were identified. For the NPs studies, special notes were recorded on the identified active constituents, the source of the NP, and how it was processed. 

## 3. Events of Detection of *A. baumannii* in the United Kingdom and Egypt

### 3.1. A. baumannii in the United Kingdom

One of the very early reports that addressed CRAB in the United Kingdom dates back to 1993 [21]. However, this study characterized a strain that was isolated back in 1985 from a blood culture in the Edinburgh Royal Infirmary. Another study referred to outbreaks in Nottingham hospital ICUs since at least 1977. However, these outbreaks were traced back to *Acinetobacter* spp. potentially including MDR *A. baumannii* [22]. In the same study, the authors characterized a more recent outbreak (1992–1993) involving MDR *A. baumannii* colonizing and infecting patients, and it was detected in the hospital environment; however, the patients with the infection remained responsive to carbapenem treatment [22]. Upon surveying the ICU of the same hospital later (1994–1995), MDR *A. baumannii* was detected in the patients and the environment, with some of the strains related to those detected earlier in previous outbreaks [23]. 

The first study reporting the detection of *A. baumannii* as one of the pathogens involved in the colonization and infection of lower airways in children who required ventilation for long-term came in 1998, but there were no reporting of the antibiotics susceptibility pattern [24]. In 1999, the clonal spread and persistence of *Acinetobacter* strains, including *A. baumannii*, was reported in a large Scottish teaching hospital, but the strains remained susceptible to imipenem [25]. 

Moving into the 21st century, one of the earliest reports from the UK was published in 2002; this study was a survey of 54 laboratories throughout the UK conducted in 2000 [26]. The *A. baumannii* complex accounted for 75% of all the isolates. The high isolation rate of *A. baumannii* complex isolates was mostly associated with lower respiratory tract infections. The carbapenems, colistin, minocycline, and sulbactam possessed the most significant activity, with 98% of the isolates remaining susceptible to imipenem. In addition, colistin has been perceived as a drug of last resort. Shortly after, CRAB that produces a class B metallo-β-lactamase was isolated for the first time in the UK from a patient who was previously hospitalized in Spain [27]. In the same year of 2002, a study on 287 *A. baumannii* isolates from 46 hospitals across the UK reported that many isolates were resistant to multiple antibiotics. However, almost all of them remained susceptible to carbapenems and colistin [28]. More intriguingly, the year of 2002 was when the first description of a CRAB outbreak in the UK was published, detailing an outbreak that took place in 1998 in a Birmingham hospital ICUs following the increase in the use of meropenem. The isolates were only susceptible to colistin, and curtains were pointed out as a potential reservoir in the outbreak [29].

An in vitro study comparing the activity of moxifloxacin and ciprofloxacin against nosocomial *A. baumannii* isolates revealed that 50.9% of the isolates were susceptible to ciprofloxacin and 60.6% were susceptible to moxifloxacin [30]. In addition, 20% of the ciprofloxacin-resistant isolates remained susceptible in vitro to moxifloxacin, while mutations in both the *gyrA* and *parC* genes were necessary for resistance to moxifloxacin in most of the isolates [30]. During an eighteen-month prospective study in an ICU in London, the mortality rate of patients with *A. baumannii* infection or colonization was found to be significantly higher than that of the patients without *A. baumannii* infection [31]. Among the isolates, resistance to gentamicin and ciprofloxacin was the most common, while the isolates remained susceptible to carbapenems.

In 2003, *Acinetobacter* strains from a hospital in Oxford were compared to those collected from three hospitals in Greece. The antibiotic resistance among the UK isolates was notably weaker than that among the Greek isolates (12% vs. 97% MDR) [32]. In addition, the Greek isolates were clonally linked, while the British ones were heterogeneous and distinguishable upon typing.

In 2004, a study was published about *A. baumannii* outbreak in a neurosurgical ICU between 1998 and 1999. It demonstrated that increasing the hospital environmental burden of *A. baumannii*, due to inadequate cleaning standards, resulted in a significant increase in patient colonization with the same strains [33]. The first report of *A. baumannii* causing skin abscesses came in the form of a case study of a neonate, where the isolated strain was sensitive to meropenem and amikacin yet resistant to amoxicillin/clavulanate, gentamicin, and cefuroxime [34].

An alarm regarding the spread of CRAB in the UK came in a study published in 2004, where 375 *A. baumannii* isolates from 24 hospitals across the UK, mainly from sputum and wound specimens, appeared to be clonally related and were MDR; furthermore, most of the isolates were carbapenem-resistant [35]. A molecular epidemiological survey of integrons in the *A. baumannii* strains in the UK indicated that class 1 integron is the most common in clinical isolates [36]. In 2006, a novel multiplex PCR was developed to detect the genes encoding the OXA carbapenemases prevalent among *Acinetobacter* in the UK [37]. The isolates belonging to the two prevalent UK *A. baumannii* ‘OXA’ clones had alleles encoding both an intrinsic OXA-51-like carbapenemase and an acquired OXA-23 carbapenemase. Contrastingly, another group of isolates had only the intrinsic *bla*_OXA-51-like_ allele, and it coexisted with the *bla*_OXA-58_ in isolates collected from one hospital. In the two later studies, the analyzed strains were mostly those previously reported [26,35]. In another study with a larger isolates pool, including some of the previously reported isolates, 627 isolates were analyzed from both OXA-23 clones 1 and 2 [38]. Both clones had *bla*_OXA-23-like_ genes, as well as the intrinsic but downregulated *bla*_OXA-51_. Whereas OXA-23 clone 1 was only susceptible to colistin and tigecycline, OXA-23 clone 2 isolates were also susceptible to amikacin and minocycline [38].

A rare, and potentially the first, incidence of *A. baumannii* infection of an aortic graft was reported in the UK in 2006, but it was responsive to antibiotic therapy combined with surgical drainage [39]. Despite being published in 2006, a study described an *A. baumannii* outbreak in a London ICU that took place between 2001 and 2002 [40]. The isolates were MDR but remained sensitive to colistin and imipenem. Since 2003, the importation of the MDR T strain has been observed in the military and non-military patients returning to hospitals in the UK from conflicts in Iraq [41]. 

In a comprehensive retrospective observational study of bacteremia in a single hospital in London between 1998 and 2006, MDR *A. baumannii* was observed to be the most frequently isolated species causing bacteremia in the ICU [42]. The carbapenem resistance soared from 0% in 1998 up to 55% by 2006. In another study on 104 *A. baumannii* isolates collected during 2006, all the isolates were from the prevalent OXA-23 clone 1. Interestingly, the efflux inhibitor 1-(1-naphthyl methyl)-piperazine (NMP) caused a reduced susceptibility (~2-fold) to tigecycline, while the same was not observed for other tetracyclines [43].

In 2008, another outbreak took place, but this time in Cambridge. All the cases were mechanically ventilated; the isolated *A. baumannii* strains were all carbapenem-resistant MDR belonging to OXA-23 clone 1 and only susceptible to colistin and tigecycline [44]. Moreover, the isolates obtained from the patients matched those from the hospital environment. In a study evaluating the Chromagar *Acinetobacter* for the detection of the enteric carriage of MDR *A. baumannii* among critically ill patients, MDR strains were prevalent among this population, either in stool or perianal swabs. The molecular characterization of these isolates indicated that they were positive for OXA-51 and OXA-23 but negative for *bla*_OXA-24_ and *bla*_OXA-58_ [45]. MDR *A. baumannii* isolates collected from 18 hospitals in and around London were analyzed, and the majority (~85%) of the isolates belonged to OXA-23 clone 1, while the rest belonged to the SE clone lineage [46]. They were all carbapenem-resistant, yet they remained susceptible to colistin and to a lesser extent to tigecycline [46].

In 2010, high-throughput whole-genome sequencing (WGS) was introduced as a new tool in epidemiological studies to analyze the spread of MDR *A. baumannii* from military casualties returning from Iraq and Afghanistan to civilians in a Birmingham hospital [47]. The new technology detected differences among the isolates that had not been identified by traditional methods and helped in achieving a better understanding of how the outbreak might have taken place.

A study reported tigecycline resistance emergence during therapy in MDR *A. baumannii* isolates that were initially susceptible only to tigecycline and colistin [48]. The isolates belonged to the epidemic UK lineage OXA-23 clone 1, and the resistance was found to be attributed to the upregulation of the AdeABC efflux pump gene.

In 2011, another report of an outbreak in a hospital in the UK described the occurrence of MDR *A. baumannii* belonging to the European clone II lineage and harboring the *bla*_OXA-51_ carbapenemase gene [49]. In 2012, *A. baumannii* strains isolated in Aberdeen were analyzed for their resistance to different antibiotics. New variant strains were identified, and, more importantly, a carbapenem-sensitive strain turned into a resistant one by acquiring the *bla*_OXA-23_ gene [50].

In 2014, a report described an outbreak of MDR *A. baumannii* in a public hospital in Birmingham, UK, that took place from 2011 to 2013 [51]. The first strain was isolated using WGS from a military patient who departed from Afghanistan. It was observed that 18 *A. baumannii* isolates did not belong to the main outbreak, while 74 isolates from 49 patients could be assigned to the same pulsotype based on genomic similarity. This study highlighted the potential benefits of using such advanced technology in investigating outbreaks.

In 2015, an epidemiological study was performed on carbapenem-resistant Gram-negative bacteria in two teaching hospitals in West London between 2009 and 2012. The rate of change in resistance among *A. baumannii* was significant and alarming, as it moved from 47% in 2009 to 77% in 2012 [52]. In 2016, a similar investigation looked into the data about CRAB, and it was indicated that their prevalence went up from 9.1% in 2011 to 31.2% in 2013 in another London hospital [53]. Epidemiological analyses of these emerging strains displayed a range of specialties and antibiograms, indicating that the seen increase was not due to a clonal outbreak. 

Reports of outbreaks occurring due to *A. baumannii* in UK hospitals became very rare after 2016. The potential reasons for this observation are discussed below. Accordingly, the first case came in 2021 in a report of a prosthetic joint with a CRAB infection that was treated in a London hospital [54]. The isolate was only susceptible to colistin and tigecycline, yet the case developed kidney injury. Accordingly, the use of cefiderocol was combined with the use of tigecycline, and the case responded well to this combination [54]. In 2021, the prevalence of the 16S rRNA methyltransferase-producing, Gram-negative bacteria was investigated in the UK. Sixteen *A. baumannii* strains were isolated in a six-month period from fourteen hospitals [55]. Eleven of these isolates combined the presence of the 16S RMTase and carbapenemase genes, mainly *bla*_OXA-23_, and they were combined with *bla*_NDM-1_ in just one case.

In 2021, a report also analyzed isolates collected between 2014 and 2018 [56]. In this collection of Gram-negative bacteria, there were only 70 *A. baumannii* strains (14.3% of 498 non-fermenters and only 3.7% of the total 1886). These numbers showed the low prevalence of *A. baumannii* in the UK compared to other European countries, where it represented 4.8% of non-fermenters and 11.6% of the total collected Gram-negative bacteria. In this pool of isolates, 87% of them were still susceptible to meropenem, and up to 94% were susceptible to cefiderocol, which was the focus of the study. All the *A. baumannii* isolates were susceptible to colistin. Interestingly, cefiderocol was still active against the carbapenem-resistant strains, highlighting its potential usefulness in treating non-responsive infections. 

Finally, the only report found in 2022 was about the initial identification of an *A. baumannii* strain expressing the novel 16S rRNA methyltransferase RmtE3, which is a new *rmtE* variant, in the UK and in the whole Europe [57]. A summary of the analyzed studies from the UK is presented in Table 1.

### 3.2. A. baumannii in Egypt

The high antibiotic pressure in Egypt and the under-regulated usage of antibiotics have caused a rapid and dreadful emergence of resistant isolates in the healthcare setting, in addition to exporting resistance to other parts of the world. Indeed, the first reports describing resistant *A. baumannii* strains that are linked to Egypt came in the form of diagnoses in patients who contracted infections in Egypt and then moved to other countries. For instance, the oldest report, published in 2008, discusses how the PER-1-producing *A. baumannii* was introduced in Hungary through a patient who was previously hospitalized in Egypt in 2006 [58]. Another example is a report in Belgium; here, a similar scenario took place, where two of the index cases were initially treated in Egypt in 2009. The two strains harbored the gene encoding the GES extended-spectrum β-lactamases of the type Bla_GES-11_ and Bla_GES-12_ and contributed to their importation to Belgium [59]. In 2011, a patient was initially hospitalized in Egypt and became infected with *A. baumannii* harboring the *bla*_NDM-1_, and it was detected upon transfer to a hospital in the Czech Republic [60]. Six days later, another strain with the same resistance pattern was recovered from another ventilated patient who shared the same room with the index case. In the same year but in Germany, the first *bla*_NDM-1_ variant was detected, and it was designated as *bla*_NDM-2_ [61]. The CRAB strain was isolated from a child who was injured in an accident in Egypt and was subsequently hospitalized there for three days before being transferred to Germany. Another clone appeared in France following the hospitalization of a patient in an Egyptian hospital, and it turned out to harbor *bla*_NDM-1_ [62].

The first report describing *A. baumannii* strains isolated and characterized in an Egyptian hospital was produced by Mohamed and Youssef in 2011 in the Northern city of Alexandria [63]. Some of the isolates were resistant to imipenem (~13%), yet they remained susceptible to tigecycline.

Another report about the prevalence of *A. baumannii* in Egypt came in 2012 from the ICUs of the Cairo University Hospitals [64]. *A. baumannii* was the most frequent cause of device-associated infections between 2009 and 2010 with ~77% of the isolates being carbapenem-resistant [64]. In the same year, an investigation into an outbreak in Assiut University Hospital in Upper Egypt detected *A. baumannii* isolates with epidemic potential in ICU patients and in the hospital environment [65].

Upon investigating the diversity of *A. baumannii* strains isolated from pediatric cancer patients in Egypt, the results indicated large genetic and epidemiological diversity among the investigated isolates [66]. Eight different *bla*_OXA-51-like_ genes were detected. Carbapenem resistance among the isolates was related to genes encoding the class-D carbapenemases (OXA-23, OXA-40, and OXA-58) [66]. In another study surveying patients in the ICU of three Egyptian hospitals, *A. baumannii* represented only 10% of the isolates; however, they demonstrated the highest percentage of carbapenem resistance (74%). All the isolates harbored the *bla*_OXA-23_ gene and the *bla*_VIM_ was identified in only 2.5% of the isolates [67]. Another study compared the efficacy of intravenous (iv) colistin vs. iv colistin combined with aerosolized colistin in treating VAP caused by MDR pathogens. MDR-CRAB was responsible for 65% of the cases, and the co-administration of the aerosolized colistin was associated with lower mortality [68].

In the city of Ismailia, *A. baumannii* represented a small proportion of the investigated isolates, yet 100% of them were MDR, 80% were extensively drug-resistant (XDR), and 60% were phenotypically carbapenemase producers [69]. In a shorter survey of only three months in two Greater Cairo hospitals, 70% of the isolated *A. baumannii* strains were carbapenem-resistant and 5% were colistin-resistant. The detected β-lactamase-encoding genes included *bla*_TEM_, *bla*_PER_, and *bla*_GES_ [70]. 

In 2015, a high prevalence of *bla*_NDM-1_ among *A. baumannii* clinical isolates in Egypt was reported for the first time [71]. In addition, the co-existence of the 16S rRNA methylase *armA* and *bla*_NDM-1_ with *bla*_OXA-23_ genes was detected among the isolates. Moreover, multilocus sequence typing (MLST) showed 27 distinct sequence types, including 11 novel ones. In the same year, the changes in the role of MDR *A. baumannii* in orthopedic surgical sites infections was investigated, and it was noted that *A. baumannii* ranked as the third most common cause by being involved in ~17% of the cases. Furthermore, none of the isolated *A. baumannii* strains were susceptible to carbapenems [72]. In another 9-month survey, *A. baumannii* represented ~23% of infections in a surgical ICU at Cairo University [73]. Surveying bacteremia infections among cancer patients for six months in 2012 indicated that MDR *A. baumannii* represented only 10% of the isolates [74].

In 2016, looking into VAP cases in Cairo University Hospital, XDR *A. baumannii* emerged as a major player (64%), and colistin was the only antibiotic that was active against all of them [75]. An investigation was carried out with the aim of determining whether a specific factor was associated with the observed increase in the acquisition of *A. baumannii*, no definite factor was identified. In 2016, MDR-CRAB was the second most frequent pathogen infecting Egyptian patients with chronic HCV who were being treated with interferon [76].

In 2017, MALDI-TOF-MS was deployed to detect carbapenemases among *A. baumannii* isolates collected from Alexandria. The technique successfully detected the disappearance of the imipenem peak with the isolates that were carbapenemase producers [77]. In the same year, the first report to come from the city of Mansoura in the Eastern Delta region reported the spread of MDR-CRAB in the ICUs of Mansoura University Hospital. Most of the isolates were only susceptible to tigecycline and colistin, with more than 95% of the isolates harboring the *bla*_IMP_ metallo-beta-lactamase gene [78]. In another study, the screening of 56 clinical *A. baumannii* isolates from cancer patients revealed a high rate of carbapenem resistance, reaching 71.4% [79]. In contrast, strains harboring the genes encoding the metallo-β-lactamases VIM and NDM-1 were highly prevalent. In addition, MDR strains also showed reduced susceptibility to chlorhexidine and cetrimide [79]. In a survey of another tertiary hospital, *A. baumannii* strains harboring *bla*_OXA-23-like_ genes were widely circulating, with carbapenem resistance reaching 100% [80]. Moreover, MLST revealed the emergence of a novel sequence type that had not previously been detected [80]. 

*A. baumannii* also emerged as the most common organism of infections following living-donor liver transplantation in Egyptian patients with cirrhosis in a study conducted in 2017 [81]. In the screening of MDR Gram-negative clinical isolates from Egypt, *A. baumannii* displayed resistance to most antibiotics, including imipenem, with the *bla_OXA-_*_23-like_ gene being highly prevalent [82]. Upon investigating the bacterial causative agent of meningitis in three different hospitals in Cairo, MDR *A. baumannii* was observed to be the most frequent Gram-negative causative agent, but the strains remained susceptible to carbapenems [83]. Looking into late-onset sepsis in a neonatal ICU in Mansoura, *A. baumannii* was observed to not be among the top three causative agents, and the collected strains were mostly carbapenem-susceptible [84]. In the same year, the examination of endotracheal aspirates in a respiratory ICU in Ain Shams University, Cairo, showed that *A. baumannii* was the most common pathogen in the cultures (26%); however, none of the strains were associated with patient mortality. All *A. baumannii* cases responded well to empiric antibiotic therapy [85].

In 2018, in the neonatal ICU of Mansura University Hospital, CRAB represented 73% of all strains during an 18-month long study [86]. Furthermore, the reduced susceptibility to tigecycline in MDR *A. baumannii* isolates in Cairo University Hospitals was investigated, and it was concluded that the amino acid mutations in the *adeS* gene in the presence of insertion IS*Aba*-1 could be responsible for the observed phenotype [87]. In the same year, a molecular investigation into the resistance of *A. baumannii* to fluoroquinolone was conducted, and it linked most of the observed resistance to mutations in the *gyrA* and *parC* genes [88]. In 2018, MDR *A. baumannii* represented ~13% of MDR Gram-negative pathogens causing bloodstream infections in Egyptian patients with febrile neutropenic cancer [89]. In a yearlong study at Cairo University, it was observed that *A. baumannii* represented 18% of surgical sites infections, but all were carbapenem-resistant [90].

CRAB collected from Zagazig hospital harbored the *bla*_OXA-23_, *bla*_NDM_, and *bla*_GES_ genes; however, while the in vitro combination of colistin and tigecycline showed a synergistic effect against them, this combination remains to be tested in vivo [91]. *A. baumannii* was then identified as the second major cause of VAP in a major University Hospital in the delta city of Tanta [92]. In the same year, there was a report of colistin-resistant CRAB strains in the ICU of Cairo University, and they were also highly resistant to other tested antimicrobials [93]. In 2018 in Mansoura, there was high prevalence of resistance to β-lactams via ESBLs and AmpC β-lactamases among the isolated *A. baumannii* strains, with the carbapenem resistance rate reaching up to 98% [94]. The screening of pan-aminoglycoside-resistant *A. baumannii* isolates in two Egyptian hospitals indicated that the resistance was mainly mediated through efflux pumps and that inhibitors of such pumps was key in enhancing the susceptibility of these isolates to aminoglycosides [95]. A study conducted in 2018 looking into ciprofloxacin resistance among *A. baumannii* strains indicated that more than 80% of the isolates were highly resistant (MIC > 32 mg/L). However, only one isolate harbored a mutation in the *qnrS* gene [96].

In 2019 and in Mansoura again, CRAB accounted for 98% of cases, with elevated levels of resistance to quinolones and aminoglycosides. The isolates remained susceptible to colistin, and their typing indicated clonality among them [97]. The molecular typing of *A. baumannii* isolated from patients with cancer in a single center in 2019 showed a high degree of multi-clonal dissemination, with five new sequence types (STs) being identified [98]. During the screening of more CRAB isolates, but this time in Alexandria, *bla*_OXA-51_, *bla*_OXA-23_, and *bla*_VIM_ were observed to be the most prevalent carbapenemase-encoding genes [99]. In another study conducted in Alexandria, but this time looking for fluoroquinolone resistance, *A. baumannii* resistance to this class of antibiotics was observed to be mainly mediated through mutations in *gyrA* and *parC*. The isolates were associated with VAP; most were XDR, and one was PDR [100]. In the same year, in a different study applying a newly developed typing method known as the PCR-based open reading frame typing (POT) method, it was demonstrated that the most common clonal linage among the tested strains was international clone 2, which was assigned the POT 122 label [101].

In 2019, MDR-CRAB isolates were prevalent among patients hospitalized in three hospitals in Cairo (16%). Most of the isolates (75%) harbored the combination of three *bla*_OXA_ genes [102]. The screening of *A. baumannii* isolates collected from patients with late-onset VAP hospitalized in both Cairo and Menoufia University hospitals showed that MDR-CRAB isolates were prevalent (84%) and that the *bla*_NDM-1_ gene was present in more than 75% of the isolates [103]. The mortality rate among the infected patients was up to 47%, and the contribution of the *bla*_NDM-1_-positive isolates to this mortality was high. By contrast, the screening of critically ill patients with chronic obstructive pulmonary disease at the respiratory ICU in Assiut University Hospital showed a low contribution of *A. baumannii* infections as opposed to methicillin-resistant *Staphylococcus aureus*. Nevertheless, CRAB represented 86% of the detected *A. baumannii* isolates [104].

In 2020, in an effort to evaluate the effectiveness of a bundle-based approach in reducing surgical site infection (SSI) in Cairo University ICUs, both patient colonization and SSI were significantly reduced, but MDR *A. baumannii* replaced *K. pneumoniae* in being the most common organism to be isolated at the end of the study as opposed to the pre-implementation stage [105]. In a retrospective study in Mansoura, the combination of tigecycline and ampicillin/sulbactam demonstrated significant clinical efficacy against XDR *A. baumannii*, although each agent was resistant alone [106]. Another study conducted in Mansoura about VAP in the ICU demonstrated that *A. baumannii* represented 11% of the cases, and 83% were CRAB [107]. Investigating an outbreak in Tanta, CRAB represented more than 80% of the isolates, with OXA-23, NDM-1 and -2, and VIM-1 and -2 carbapenemases being responsible for this type of resistance [108]. In a survey of febrile neutrophilic patients in two hospitals in Cairo, *A. baumannii* represented 37% of the isolates and 98% were CRAB [109]. For the first time, a study examining community-acquired infections in Egypt noted that *A. baumannii* represented about 11% of the infections, and the levels of carbapenem resistance among them were high (~50%), yet lower than those previously reported for hospital-related infections. However, there were both MDR and XDR strains among the collected isolates [110].

In 2020, the first study on *A. baumannii* infection in Upper Egypt was conducted, and it was also the first to conduct a survey with the aim of identifying co-infections during the COVID-19 pandemic. *A. baumannii* was observed to be among the detected infections and represented about 17%, with almost 70% of them being resistant to imipenem and *bla*_NDM_ being present in all of them [111]. In another study, healthcare-associated infections were assessed in different setting, and *A. baumannii* accounted for 19% of all infections, with CRAB accounting for around 50% of them, yet colistin resistance was up to 20%, which is very alarming [112]. A more alarming report came in the same year, and this time using WGS for the first time, where colistin resistance was observed to have reached up to 53%. Molecular typing of the isolates indicated that the majority belonged to ST158, representing a clone circulating in Mediterranean/Middle Eastern countries [113]. In a related study, colistin resistance was observed to be up to 41% [114]. Upon investigating isolates collected in Fayoum, it was revealed that 63% of the *A. baumannii* isolates were CRAB, and both *bla*_OXA-23-like_ and *bla*_OXA-24-like_ were the most prevalent carbapenemase-encoding genes [115]. Contrastingly, in Minia, in the Upper Egypt region, carbapenem resistance was relatively low (20%); however, genes encoding extended spectrum β-lactamase (ESBL) and metallo-β-lactamases (MBLs) were spread among them [116]. Among the *A. baumannii* isolates collected from an ICU in Ain Shams University, 14% harbored the *mcr-1* gene encoding colistin resistance and were resistant to the last-resort antibiotic, yet 85% were CRAB [117].

In 2021, two studies focused on resistance to aminoglycosides among Egyptian *A. baumannii* isolates from Alexandria and Ismailia. The rate of resistance was up to 82% in the former and 67% in the later, but the predominant resistance gene was different in the two settings: *aphA6* in Alexandria and *aacC1* in Ismailia [118,119]. In the nearby city of Zagazig, the relationship between the capability of biofilm formation and antibiotics resistance among *A. baumannii* isolates was investigated, and it was observed that biofilm-producing MDR and XDR isolates were highly prevalent [120]. In a survey of patients with cancer infected with CRAB for the co-existence of more than one carbapenemase gene, it was found that more than 94% of the isolates harbored more than one gene, with *bla*_NDM_ being the most prevalent [121]. Returning to Cairo University ICUs, it was observed that the CRAB isolates were very well spread, and *bla*_OXA-23_ turned out to be the most prevalent carbapenemase-encoding gene followed by *bla*_NDM_ [122]. Looking into the prevalence of bacterial and fungal infections during the second wave of COVID-19, MDR *A. baumannii* ranked second as the most common cause (27.4%) of infection among mechanically ventilated patients [123]. In another study, the rate of XDR isolates among *A. baumannii* isolates reached 68%, while it was 2% for PDR isolates [124].

In 2021, a study examined antibiotics resistance, in vitro biofilm formation, and the in vivo virulence of MDR *A. baumannii* clinical isolates [125]. Alarmingly high proportions of strong biofilm-forming strains were observed, and they were also associated with high virulence in the in vivo model. In another study, WGS was used to characterize XDR *A. baumannii* from Egypt and demonstrated linked genes and diverse mobile genetic elements presenting novel insertion sites and genetic organizations. Moreover, the emergence of *bla*_ADC-257_ was recorded [126]. Applying WGS again but on a larger scale and on isolates collected from pediatric patients with cancer identified the genetic basis of the extensive antibiotic resistance, including the last-resort drug, colistin. Furthermore, the detection of CRISPR/Cas was associated with the failure of phage therapy [127]. Similar findings were obtained upon analyzing more isolates from Alexandria via WGS; the *bla*_OXA-23_ genes were predominant among the isolates, but they were located on various mobile elements [128]. In 2022, another study employed WGS on Egyptian MDR and XDR *A. baumannii* isolates and demonstrated the diversity among the isolates and different genetic organizations of resistance islands carried by those strains [129]. A summary of the analyzed studies from Egypt is presented in Table 2.

## 4. Efforts in the United Kingdom and Egypt to Combat *A. baumannii* Using Natural Products

The first report from the UK about exploring the role of NPs in fighting MDR *A. baumannii* was published in 2014. The authors demonstrated that curcumin has MIC of 256 µg/mL against *A. baumannii*, and epigallocatechin gallate (EGCG) was observed to display activity ranging from 128 to 1024 µg/mL on different strains [131]. Despite this little antibacterial activity of the curcumin alone, upon combination with the EGCG, the antibacterial activity was significantly enhanced, reaching an MIC of 4 µg/mL. In another study, the antibacterial effects of polymyxin B and curcumin and their combination against MDR bacteria associated with traumatic infections were investigated. The MIC of polymyxin B was significantly reduced by curcumin (3- to 10-fold), indicating a synergistic effect. Time-killing assay indicated the bactericidal effect of the combination. Moreover, the cytotoxicity of curcumin was reduced in the presence of polymyxin B [132].

In 2017, it was observed that the engineered honey SurgihoneyRO (SHRO), in addition to having good antibiofilm activity, can effectively reduce the seeding of *A. baumannii* biofilms [133]. In another comparative study, the polyphenols theaflavin and epicatechin alone and showed a bacteriostatic effect against *A. baumannii*; however, upon testing the combination, they showed bactericidal activity, and they were also very effective in the in vivo model when using *Galleria mellonella* [134].

From an examination of the Egyptian studies investigating the potential role of natural products in fighting *A. baumannii*, the first report, which was published in in 2017, showed the flavonoids and phenolic compounds isolated from manuka honey were very effective in the treatment of wound infection upon incorporation into a hydrogel. The principal active components for this activity were luteolin, isoferulic acid, kaempferol, and chrysin [135]. In the same year, the volatile oils (VOs) of the anise and star anise fruits were obtained via hydrodistillation. In addition, following distillation, the remaining water samples of both plants were freeze dried and tested for their antibacterial effect against Gram-positive and -negative bacteria, including *A. baumannii*. The phenolic contents of waste products were determined using HPLC. Star anise waste showed more activity than anise waste, which may be attributed to its higher phenolic content. In addition, a synergistic effect between both plants and antibacterial drugs was detected [136].

In 2018, another study reported that ripened fruits of *Schinus terebinthifolius* contain active ingredients that have good antimicrobial activity against *A. baumannii* [137]. The most active fraction was the acetone and its main components were oleic acid (38.7%), α-phellandrene (13.3%), δ-cadinene (11.1%), and linolenic acid methyl ester (6.6%) [137]. Two other studies about VOs were published in 2018. In the first one, ten VOs were tested for their effects on MDR *A. baumannii* [138]. The selected plants are commonly used in Egyptian folk medicine. Among the tested oils, clove, thyme, and rosemary were the most active oils, followed by marjoram, black seed, lemongrass, fennel, peppermint, chamomile, and anise. In the second study, eight VOs were tested against the Gram-negative bacteria *Acinetobacter baumannii* and *Klebsiella pneumoniae* [139]. The tested oils were obtained from some Lamiaceae plants via hydrodistillation; *Origanum majorana* L. (two samples), *Origanum syriacum* L., *Thymus capitatus* L., *Thymus vulgaris* L., *Salvia fruticosa* Mill., *Mentha virdis* L., and *Lavandula officinalis L.* were among the tested oils. *T. capitatus*, *T. vulgaris*, *O. majorana* from two localities, and *O. syriacum* were proved to be the most effective oils. The composition of tested oils was investigated using GCMS.

In 2020, a study showed that an aqueous extract of *Acacia nilotica* had good antimicrobial activity against CRAB, but it did not interfere with its biofilm formation capabilities as seen with *E. coli* and *K. pneumoniae*. The main components that could be linked to the observed activity were 3-cyclohexane-1-carboxaldehyde-2,6,6-trimethyl; á-selinene (CAS); oleic acid; and globulol [140]. In the same year, another group studied the potential antimicrobial activities of *Pimenta dioica* and *Pimenta racemosa* essential oils against *A. baumannii* [141]. The essential oils demonstrated good activities in killing the microorganism, preventing biofilm formation, and eradicating already formed biofilms. Moreover, upon testing in an animal model of skin infection, it was observed that they significantly decreased the microbial burden in skin lesions. Analysis of the main components indicated the predominance of monoterpene hydrocarbons, with β-myrcene as the major identified component [141]. In another study, the aqueous methanolic (50%) extract of star anise was tested against MDR *A. baumannii* for killing, biofilm inhibition and detachment. The extract demonstrated significant activities in all three assays [142]. The observed activities were also extended to the Gram-positive pathogen *S. aureus*, and they were related to the phenolic acids and flavonoids contents of this plant. In a very similar study, an extract of *Caralluma quadrangular* was tested against both MDR *A. baumannii* and *S. aureus*. The extract showed good activities in the same three phenotypes described earlier, but the isolated potential compounds from the fractions were not tested further against *A. baumannii*; rather, they were tested against *S. aureus* [143].

Honey products, including propolis, venom, and wax, are NPs that are commonly used against bacterial infections. The MIC and MBC of bee products against *Pseudomonas* and *A. baumannii* were assessed in a previous study. Propolis revealed the most potent activity against *P. aeruginosa* and *A. baumannii*, while bee venom showed the least activity [144]. In another report, the effect of beehive air on *S. aureus*, *K. pneumoniae*, *A. baumannii*, and multidrug-resistant *S. aureus* was investigated. Beehive air is a well-known remedy for bronchitis, lung fibrosis, and asthma. The constituents of beehive air were determined through headspace solid-phase microextraction gas chromatographic analysis (HS-SPME GCMS). Beehive air showed superior antibacterial activity to that of its individual components, i.e., bee venom, wax, and honey [145].

A different approach, namely, the in silico docking of herbal plants to potential targets, with the aim of gaining a better understanding of how active ingredients from natural products could be working against *A. baumannii*, has been used [146]. The authors demonstrated good antimicrobial activity of *Syzygium aromaticum* against *A. baumannii*, and they showed that guanosine displays fitting to the binding site of *A. baumannii* PBP1 and/or PBP3 with the highest energy in a comparable way to that of imipenem [146]. In another study, both cinnamic acid and gallic acid were tested against MDR *A. baumannii*. Both showed promising activities, with cinnamic acid having a lower MIC (1.2 mg/mL vs. 1.65 mg/mL). Moreover, both natural compounds showed very good activity in preventing biofilm formation upon use in sub-MIC concentrations [147]. In continuation of the former study, another investigation showed a significant reduction in resistance upon combining cinnamic or gallic acid with imipenem, amikacin, or doxycycline [148]. Moreover, on the molecular level, upon exposure of strong-biofilm-forming *A. baumannii* to gallic acid, there was a significant downregulation in the expression of the *bap*, *csuE*, and *ompA* genes, which are involved in biofilm formation [148]. Finally, the ethanol extracts of *Nigella sativa* and *Lawsonia inermis* were tested against the following Gram-negative bacteria: *P. aeruginosa*, *K. pneumoniae*, and *A. baumannii*. Both extracts showed various activities against all tested microorganisms. A nanoformulation of *N. sativa* showed a greater effect than the plant extract on both *A. baumannii* and *K. pneumoniae*, while no activity was detected on *P. aeruginosa* [149].

A list of the tested natural products against *A. baumannii* in studies from the UK and Egypt is presented in Table 3.

## 5. Discussion

After decades of being observed as a harmless microorganism, *A. baumannii* has taken the center stage as a superbug that is harming healthcare settings everywhere. Its notorious ability to acquire antibiotic resistance in addition to its possession of multiple virulence factors formed a recipe for a formidable pathogen that can easily prey on vulnerable immunocompromised individuals [150]. In our attempt to trace back the incidence of the initial infections related to *A. baumannii* in the UK and Egypt, a big gap was observed. Reports indicate that *A. baumannii* could have been involved in UK infections as early as 1977. Although almost 30 years later, we observed the emergence of multiple reports of incidences where foreigners were diagnosed with *A. baumannii* strains that they most probably acquired while being in Egypt, the first report from Egypt was published in 2011 [63].

A reason for this observation and this gap between the two countries in reporting *A. baumannii* infections could be the more accurate and advanced diagnostic resources in the UK than those in Egypt. This is most probably true as *A. baumannii* is a ubiquitous microorganism in the hospital environment. The later observation was manifested with the surge seen in *A. baumannii* infections following the beginning of military operations in Iraq in 2003 [151]. Epidemiological tracing of such injuries indicated that the most likely sources for these strains are the inanimate surfaces in field hospitals, as they were linked to those strains isolated from the injured soldiers [152].

**Figure 2:**
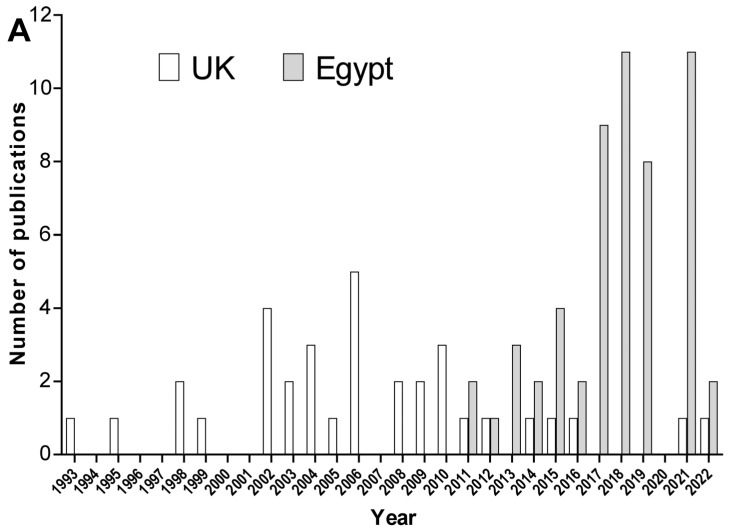

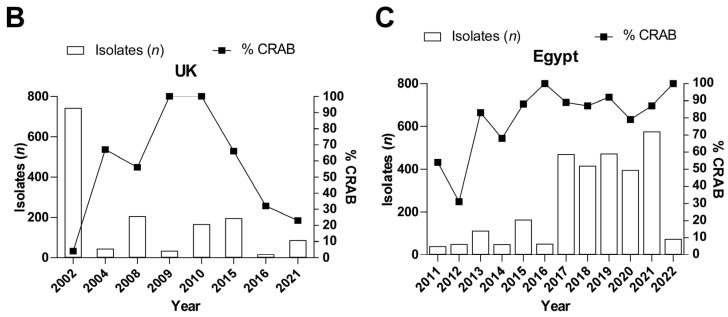
The trends of *A. baumannii* data in both the UK and Egypt. (**A**) Comparison of the frequency of *A. baumannii* studies in PubMed and Web of Science from the UK (open bars) and Egypt (gray bars) from 1993 to 2022. (**B**) The trends in the changes in the number of isolates detected per year (open bars) and the rate (%) of CRAB isolates within those collected (black boxes) in the UK. (**C**) The trends in the changes in the number of isolates detected per year (open bars) and the rate (%) of CRAB isolates within those collected (black boxes) in Egypt. The studies that were included in B and C are those that covered at least 10 isolates and have reported data about carbapenems susceptibilities.

Interestingly, as we entered the second decade of the 21st century, reports of *A. baumannii* infections and its characterization started to pick up in Egypt, while they dropped dramatically in the UK (Figure 2A). This could be explained by the fact that by this time, new technologies became more available to Egyptian researchers, such as the Vitek system for identification and susceptibility testing, PCR, and MALDI-TOF-MS, so it became easier for them to be deployed for such studies. On the other hand, the sharp decrease in the number of publications describing incidences of *A. baumannii* infection in the UK is most probably due to the tendency of researchers in the UK to conduct more sophisticated research than simply reporting on the detection of a certain pathogen in the hospital setting. Recording such information became a routine practice to keep track of infections in hospitals and to take appropriate actions rather than publishing the data in a research article. Accordingly, the incidence reporting proportion of all the studies linked to both the UK and *A. baumannii* accounted for only 37 out of 654 (5.7%), while in the case of Egypt, it accounted 73 out of 299 (24.4%). However, we should not neglect the fact that reports comparing incidences of infections with resistant pathogens in the UK with other countries, such as those in the European Union (EU), indicated that the UK indeed has lower rates [56]. 

Looking at the data from another point of view, following the trends in reporting the detection of CRAB, it is obvious that in the case of the UK, there was a gradual increase in the % of CRAB that peaked at the beginning of the second decade of the 21st century; then, it started to decline dramatically (Figure 2B). On the other hand, in the case of Egypt, despite the late start of detection, there was a gradual increase in the reported percentages of CRAB until around 2015/2016; then, the CRAB% remained high (~80% and above) (Figure 2C). These findings are very alarming in regard to the situation in Egypt, and immediate and swift interventions to contain such a threat are required. 

In addition, there is a difference between the two countries in the number of reported strains over the years; the UK started with very high numbers, and then it declined (Figure 2B), reflecting the decrease in the number of incidence reports (Figure 2A). Contrastingly, the number of isolates reported from Egypt was initially lower and began to increase later; however, a continuous increase was observed over the years, and the number of isolated reports from Egypt remains high, with a peak in the year 2021 (Figure 2C).

The threat of antimicrobial resistance (AMR) has been recognized globally and countries have responded differently, either in the timing or the scale of the response. For instance, the UK was one of the first countries to initiate a National Action Plan (NAP) on AMR as early as 2000 [153]. This was even before the Global Action Plan (GAP) endorsed by the member states the WHO member states in 2015 [154]. 

Multiple measures have been put in place to face this growing threat, they focused on different areas including increasing the awareness and understanding of the aspects of AMR, preserving the available treatments, and encouraging the development of newer ones. Strict measures are implemented in the UK regarding the prescription and the use of antibiotics in clinical practice. Also, infection control measure in healthcare institutions is tightly regulated. In addition, resources to promote the discovery of new and alternative antimicrobials are available [153].

Nevertheless, the latest NAP highlights that are more actions need to be taken. These actions especially regarding the percentage of the inappropriate antibiotic prescription and the prescribing rates in the UK being higher than that recorded in other parts of northern Europe [155].

On the other hand, Egypt has set its first NAP on AMR in 2018 and it is extended to 2022 [156]. Prior to setting this plan, the extent of AMR in Egypt was aggravated with multiple practices that led to the widespread of resistance among pathogens either in the hospital settings or in the community. For instance, antibiotics are purchased without prescription. Physicians frequently prescribe antibiotics in conditions that do not warrant their use, and pharmacists do the same even in higher rate (81% vs. 64%) [157]. Resistance is soaring in hospitals to a wide range of antibiotics unfortunately including carbapenems in pathogens like *A. baumannii* as seen in the studies discussed in the current review. Moreover, antibiotic prescribing quality indicators showed that the application of the documentation rate of the STOP/review dates and reasons of using antimicrobials is less than 50% except in ICU [156]. Also, the use of antibiotics in animals in sub-therapeutic doses is also adding to AMR in Egypt, for instance MDR pathogens have been detected in animal products in milk products [158]. 

The Egyptian NAP has set the goals that by the end of 2022, there would be a 10% reduction in AMR-related deaths, use of antibiotics in animals for growth promotion, use of antibiotics for plants protection, and total use of antibiotics, and a 20% reduction in incidences of infections due to drug resistant pathogens. This plan could have been hampered somehow due to the COVID-19 pandemic that hit the world late in 2019 and its health- and economy-related consequences. For sure the scale of such negative impacts has been more pronounced in countries with lower economic levels. Nevertheless, setting such action plan for sure is a good step towards successful containment of the AMR crises in Egypt. 

The implications of differences in the application of infection control measures especially restrictions in antibiotics usage have been demonstrated long ago. For instance, in 1998, a comparative study was conducted comparing *A. baumannii* isolates from an ICU in Nottingham, UK and another one in Soweto, South Africa [159]. Fingerprinting experiments indicated that a single MDR *A. baumannii* strain was the main player in UK setting for 11 years. Strict infection control measures could have selected for the single clone to colonize the hospital environment and causing infections among patients occasionally. On the other hand, the South African isolates formed unrelated molecular clusters and exhibited different antibiograms. The less restricted usage of antibiotics in the South Africa setting, has resulted in the development of multiple strains with a variety of resistance levels to a wider range of antimicrobials [159]. Twenty-five years later, we can still see the evidence that these differences can still be reflected on the variations in the trends and resistance levels observed between the UK and Egypt as demonstrated in the current review. 

Another gap between the two countries is the application of advanced technologies. The first study from the UK using WGS to characterize *A. baumannii* strains was published in 2010, while the first study from Egypt using a similar approach was published ten years later, in 2020 [47,113]. Of course, the time gap here is much shorter than the one discussed above, but it is still big.

**Figure 3:**
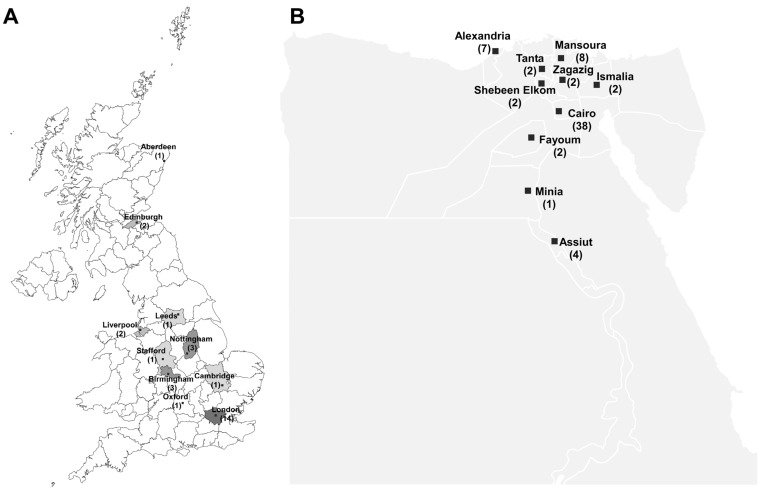
Geographical distribution of the studies in the UK (**A**) and Egypt (**B**). The map of the UK was generated using MapChart (https://www.mapchart.net/uk.html, accessed on 17 December 2022), while the map of Egypt was obtained from the freely available website https://www.slideegg.com/egypt-powerpoint-free (accessed on 30 November 2022). Studies including isolates whose sources were not clearly indicated, those that had more than two localities, and those with origins outside the UK and Egypt were not included in the tally. If a study involved isolates from two localities, it was counted as a study for each of them.

One point of interest upon comparing the data from the two countries is the geographical distribution of the source of the isolates characterized in the different studies (Figure 3). Most of the isolates in the two countries came from the capitals and their surroundings: London (14 reports) and Cairo (38 reports). However, the first reports were from regions outside the capital: Edinburgh in the case of the UK and Alexandria in the case of Egypt. Following those, reports started to come more frequently out of the capitals and other cities throughout the respective countries. The distribution of the reports among different localities within the same country is linked to the availability of surveillance and detection tools. For instance, in case of Egypt, the two cities that followed Cairo in the frequency of reports were Mansoura and Alexandria, which are big cities with good healthcare infrastructures.

In addition to the detection of *A. baumannii* as a causative agent of infection, determining how resistant strains are to the currently used antibiotics, especially carbapenem, is equally important. Excluding the studies intentionally working on CRAB, many of the studies reported from Egypt included higher percentages of CRAB than those from the UK (Table 1 and Table 2). Similar trends can be seen in the proportions of MDR isolates. A major factor contributing to such differences is how tight the administration of antibiotics is controlled. Unfortunately, in Egypt many of the medications, including antibiotics, are dispensed and administered without prescription. For instance, according to a European report, it was estimated that the proportion of self-medicated antibiotics use in Egypt could be as high as six-fold [160]. 

Natural products have been a contributor to many drugs of diverse indications, including antimicrobial therapy [161]. Thus far, there is a growing trend of declining interest in investigating NP resources for drugs, especially among major pharmaceutical industries in the developed world [162]. This trend is attributed to the technical barriers for screening, isolation, characterization, and optimization [163]. However, the introduction of new technologies, such as metagenomics, genome minding, combinatorial chemistry, and high-throughput screening, is helping in reviving the interest in NPs, particularly as antimicrobials [162,163]. Due to the difficulties faced in accessing these technologies in Egypt, researchers in Egypt have focused more of their attention on simple traditional screening approaches. This is also reflected in the depth of the analyses conducted on such NPs.

The screening NPs for effective antimicrobial agents against MDR *A. baumannii* is not limited to the UK and Egypt. Studies from other countries found sources other than those reported here. For instance, EGCG from green tea was observed to display a significant antibacterial effect against *A. baumannii* clinical isolates with MICs as low as 0.078 mg/mL [164]. A mixture of VO constituents, including eugenol, carvacrol and cinnamaldehyde, proved to be active against *A. baumannii* in vitro and in vivo [165]. Furthermore, norwogonin isolated from *Rosa rugosa*, *Scutellaria baicalensis*, and *Terminalia chebula* was highly active against *A. baumannii* [166]. Finally, the inhibitory effect of *Syzygium aromaticum* (clove), *Nigella sativa* (black cumin), *Commiphora molmol* (myrrh), and *Allium sativum* (garlic) on *A. baumannii* was studied, and the ethanoic extract of clove showed the most potent activity [167]. More in depth analyses of the identified moieties are needed to develop these findings into effective commercial therapeutics to be used in clinical practice.

NPs have always been considered as safer alternative to synthetic drugs. However, this idea has been proven to be faulty. NPs are always exposed to contaminations caused by mycotoxins, such as aflatoxins, and heavy metals, including lead, mercury, and arsenic, which have severe adverse effects on human health [168]. EGCG, the main phenolic in green tea, was reported to be hepatotoxic in high doses. Moreover, high doses of green tea itself have demonstrated significant hepatoxic effects [168]. VOs are one of the most used NPs discussed in the current article to tackle MDR *A. baumannii*. Despite their wide applications and uses, their safety profile is not fully studied. Their complex nature is one of the main reasons for the difficulty in identifying the responsible component(s) for their unwanted effect. Although VOs are generally considered safe, some intoxication incidences have been reported. For instance, in the US, 966 intoxication cases from tea tree oil in children aged up to 6 years old were reported. Furthermore. in Australia, 1387 cases were reported for VO intoxications between 2014 and 2018 [169]. Photosensitization upon topical application is considered one of the common adverse effects of aromatherapy. Data have also been reported on neurological effects, endocrine disrupting potential, and abortifacient adverse effects of NPs [169].

Accordingly, establishing partnerships between researchers in high- and low-income countries could be helpful in filling the gaps identified in this review. These could involve both surveillance levels and research on NPs. These partnerships could be in the form of funding to support infrastructure or the dissemination of technical expertise. This should be reflected in promoting a certain quality of research, especially in LMICs such as Egypt.

A limitation of the current review is that the literature review was restricted to only the PubMed and Web of Science databases. Therefore, we might have missed one or more of the relevant reports. However, the fact that studies included in this database are known for their scientific quality and rigorousness should add to the value of the current report. Expanding the search to other databases and maybe including other countries could be addressed in a future study. 

## 6. Conclusions

*A. baumannii* infections were detected in the UK long before they were observed in Egypt. However, the overall number of studies from Egypt addressing either the incidence of infections or the testing of NPs against *A. baumannii* is much higher than that from the UK. These differences were attributed to the difference in the available resources for research and the level of detail to which this research could be conducted. On the other hand, the observed differences in the number of reported incidents and the level of resistance especially to carbapenems with Egypt being a larger contributor is most likely related to the strictness in the application of infection control measures. Tighter controls on antibiotics usage are highly recommended to help curb this threat. Although some of the identified NPs demonstrated superior activities against *A. baumannii* in vitro and in vivo, either as antimicrobials or even antivirulence agents, extending their potential to clinical practice remains a difficult task. This could be due to some of their potential adverse effects and/or lack thereof, as well as their complexity. Effective partnerships between countries of different economic levels could help fill such gaps and promote better surveillance and identification of potential new therapeutics using NPs that can hopefully reach the clinical application stage.

## Figures and Tables

**Table 1:** *A. baumannii* studies from the United Kingdom.

Study	Isolates (*n*)	MDR%	CRAB %	Isolates Characterization	Genes	Reference
Paton et al., 1993	1	100	100	AST by Stokes	-	[21]
Crowe et al., 1995	11	100	-	API 20NE, PFGE, ribotyping	-	[22]
Webster et al., 1998	6	100	-	API 20NE, RAPD	-	[23]
Morar et al., 1998	-	-	-	-	-	[24]
McDonald et al., 1999	18	-	-	API 20NE, PFGE	-	[25]
Henwood et al., 2002	443	-	2	PCR, tRNA spacer fingerprinting	*bla*_IMP_, *bla*_VIM_, *bla*_OXA-23_, *bla*_OXA-24_	[26]
Towner et al., 2002	1	100	100	tRNA spacer fingerprinting, Etest, PCR	*bla*_IMP_	[27]
Spence et al., 2002	287	-	1.8	tDNA fingerprinting, RAPD	-	[28]
Das et al., 2002	13	100	100	API 20NE, PFGE	-	[29]
Spence et al., 2003	226	-	-	tDNA and AFLP fingerprinting, Etest. PCR	*gyrA*, *parC*	[30]
Theaker et al., 2003	27	-	-	PCR, DNA sequencing	-	[31]
Dimopoulou et al., 2003	17	12	-	PCR, DNA sequencing, REP-PCR	16S rDNA	[32]
Denton et al., 2004	27	0	0	API 20NE, AST by Stokes, PFGE	-	[33]
Ng et al., 2004	1	100	0	AST	-	[34]
Turton et al., 2004	375 *	100	67	API 20NE, tDNA fingerprinting, PFGE, Etest, PCR	*bla*_IMP_, *bla*_VIM_, *bla*_OXA-23_, *bla*_OXA-24_	[35]
Turton et al., 2005	-	-	-	PCR, PFGE, DNA sequencing	*bla*_OXA-23_, Class 1 and 2 integron cassettes	[36]
Woodford et al., 2006	168	-	-	Multiplex PCR	*bla*_OXA-51_, *bla*_OXA-23_, *bla*_OXA-58_, *bla*_OXA-24_	[37]
Coelho et al., 2006	627	-	-	PFGE, PCR	*bla*_OXA-51_, *bla*_OXA-23_	[38]
Pencavel et al., 2006	1	-	-	-	-	[39]
Wilks et al., 2006	136	100	0	API 20NE, PFGE	-	[40]
Turton et al., 2006	25	-	-	PFGE	Integron cassettes	[41]
Wareham et al., 2008	187	34	52	API 20NE, AST by BSAC DD	-	[42]
Bean et al., 2009	104	-	-	API 20NE, AST by BSAC DD, PCR	*bla*_OXA-23_	[43]
Enoch et al., 2008	19	100	100	PFGE, AST by BSAC DD	-	[44]
Gordon and Wareham, 2009	34	100	-	CHROMagar, PCR	*bla*_OXA-51_, *csuE*, *ompA*	[45]
Livermore et al., 2010	166	100	100	PFGE	*bla*_OXA-23_	[46]
Lewis et al., 2010	6	100	100	Vitek 2, PCR, VNTR, PFGE, WGS	Whole genome	[47]
Hornsey et al., 2010	9	100	100	API 20NE, PCR, Etest, RT–PCR	*bla*_OXA-51_, *bla*_OXA-23_, *adeAB*	[48]
Adams et al., 2011	3	100	-	PFGE	*bla*_OXA-51_	[49]
Lopes et al., 2012	9	-	11	PCR, DNA sequencing, PFGE	*bla*_OXA-51_, *bla*_OXA-23-like_, *bla*_OXA-40-like_, *bla*_OXA-58-like_, *bla*_OXA-143-like_, *bla*_ADC_, *gyrA*, *parC*, Class I integrons	[50]
Halachev et al., 2014	112	100	-	Vitek 2, PFGE, WGS	Whole genome	[51]
Freeman et al., 2015	196	100	65.8	-	-	[52]
Hughes et al., 2016	16	-	31.2	Vitek 2	-	[53]
Mabayoje et al., 2021	1	100	100	WGS	Whole genome	[54]
Taylor et al., 2021	16	100	69	MALDI-TOF-MS, PFGE, WGS	*armA*, *bla*_OXA-23_, *bla*_NDM-1_	[55]
Gant et al., 2021	70	-	13	MALDI-TOF-MS	-	[56]
Taylor et al., 2022	1	100	100	PCR, WGS	*rmtE3*, *bla*_OXA-65_, *bla*_OXA-72_	[57]

Abbreviations: Amplified fragment length polymorphism (AFLP), Antibiotic susceptibility testing (AST), British Society for Antimicrobial Chemotherapy Disc Diffusion (BASC DD), Matrix-assisted laser desorption/ionization-time of flight mass spectrometry (MALDI-TOF-MS), Polymerase chain reaction (PCR), Variable number tandem repeat (VNTR), Pulsed-field gel electrophoresis (PFGE), Random amplified polymorphic DNA (RAPD), Repetitive extragenic palindromic (REP-PCR), Reverse transcription polymerase chain reaction (RT-PCR), Whole genome sequencing (WGS). “-” indicates that the data was not mentioned or clearly inferred from the data in the report. * 45 isolates only were analyzed for AST.

**Table 2:** *A. baumannii* in Egypt.

Study	Isolates (*n*)	MDR%	CRAB%	Isolates Characterization	Genes	Reference
Szabó et al., 2008	1	100	0	VITEK 2, PCR, IEF	*bla*_PER-1_, *bla*_TEM-1_	[58]
Bogaerts, et al., 2010	2	100	100	VITEK 2, MALDI-TOF-MS, AST by KB, PCR	*bla*_GES-11_, *bla*_GES-12_, *bla*_OXA-82_, *bla*_OXA-94_	[59]
Hrabák et al., 2012	2	100	100	API ID32 GN, MALDI-TOF-MS	*bla*_NDM-1_	[60]
Kaase et al., 2011	1	100	100	API 20NE, AST by KB, PCR, MLST	*bla*_NDM-2_	[61]
Bonnin et al., 2013	1	100	100	16S rRNA gene sequencing, PCR, MLST	*bla*_NDM-1_	[62]
Mohamed and Youssef, 2011	15	-	13	API 20NE	-	[63]
El-Kholy et al., 2011	26	-	76.9	Conventional methods	-	[64]
Soliman et al., 2012	51	61	31.2	API 20 NE, AST by KB, CDT, PCR	*bla*_OXA-51_, Class I integrase	[65]
Al-Hassan et al., 2013	34	-	73	VITEK 2, Phoenix, PCR, DNA sequencing, PFGE, MLST	*bla*_OXA-51_, *bla*_OXA-23_, *bla*_OXA-40_, *bla*_OXA-58_, *bla*_OXA-64_*bla*_OXA-65_, *bla*_OXA-66_, *bla*_OXA-69_, *bla*_OXA-71_, *bla*_OXA-78_, *bla*_OXA-94_, *bla*_OXA-89_	[66]
Fouad et al., 2013	39	80	74	MHT, IPD, PCR, ERIC-PCR	*bla*_OXA-51_, *bla*_OXA-23_, *bla*_VIM_, *int1*	[67]
Amin et al., 2013	40	100	100	AST by KB, Etest	-	[68]
Nageeb et al., 2014	10	100	60	API 20NE, AST by KB, MHT	-	[69]
Al-Agamy et al., 2014	40	100	70	API 20NE, PCR	*bla*_TEM_, *bla*_PER_, *bla*_GES_	[70]
El-Sayed-Ahmed et al., 2015	150	-	87.3	MALDI-TOF-MS, PCR, AST by KB, Vitek 2, MLST	*bla*_OXA-51_, *bla*_OXA-23_, *bla*_NDM-1_, *armA*	[71]
Helal et al., 2015	15	100	100	CHROMagar, PCR	*bla*_OXA-51_	[72]
Ghaith et al., 2015	54	-	-	CHROMagar, PCR, MALDI-TOF-MS	*bla*_OXA-51_	[73]
El-Mahallawy et al., 2015	8	-	-	Microscan, AST by KB	-	[74]
Hasanin et al., 2016	30	100	100	API 20NE, E test	-	[75]
El-Kholy et al., 2016	22	100	100	MicroScan, Biolog Microlog, AST by KB	-	[76]
Abouseada et al., 2017	50	-	78	PCR, MALDI-TOF-MS	-	[77]
Alkasaby and Zaki, 2017	280	100	95.7	Etest, PCR	*bla*_OXA-51_, *bla*_TEM_, *bla*_SHV_, *bla*_CTX-M_, *bla*_IMP_, *bla*_SIM_, *bla*_GIM_,	[78]
Gomaa et al., 2017	56	88	71.4	Vitek, PCR	*bla*_OXA51_, *intl1*, *bla*_VIM_, *bla*_NDM-1_, *qacE*, *qacEΔ1*	[79]
Ghaith et al., 2017	50	100	100	PCR, MLST	*bla*_OXA-51_, *bla*_OXA-23_	[80]
Montasser et al., 2017	19	100	60	-	-	[81]
Helmy and Kashef 2017	15	86.6	66.7	API 20NE, PCR	*bla*_OXA-23_, *aac-Ib*, *bla*_TEM-1_, *bla*_CTX-M-15_	[82]
Abdelkader et al., 2017	7	100	0	AST by KB, PCR	*bla*_CTX-M_, *bla*_SHV_, *bla*_TEM_, *qnr*	[83]
Nour et al., 2017	6		17	AST by KB, CNPt		[84]
Todary et al., 2017	16	-	-	API 20NE, Vitek 2	-	[85]
Sultan and Selim, 2018	124	94.5	73.4	API 20NE, AST by KB	-	[86]
Hassan et al., 2018	63	100	-	PCR, AST by KB, Etest	*bla*_OXA-51_, *adeR*, *adeS*, *adeB*	[87]
Zaki et al., 2018	140	100	100	RFLP-PCR	*gyrA*, *parC*	[88]
Tohamy et al., 2018	12	100	83	Microscan, AST by KB, PCR	-	[89]
El-Kholy et al., 2018	6	100	100	VITEK 2, Etest, MALDI-TOF-MS, PCR	*bla*_VIM_	[90]
Ramadan et al., 2018	50	-	60	VITEK 2, AST by modified KB, PCR	*bla*_OXA-23_, *bla*_NDM_, *bla*_GES_	[91]
Abd-Elmonsef et al., 2018	9	100	33	AST by KB	-	[92]
Abdulzahra et al., 2018	40	100	100	Vitek 2, PCR	*bla_OXA-51_*, *bla_OXA-23_*, *pmrCAB*	[93]
Said et al., 2018	50	98	98	AST by KB, PCR, ERIC-PCR	*bla*_TEM_, *bla*_PER_*, bla*_SHV_, *bla*_VEB,_ *bla*_ADC_	[94]
Moustafa et al., 2018	57	-	-	PCR, AST by KB	-	[95]
Hamed et al., 2018	23	-	-	Vitek 2, PCR, DNA sequencing, ERIC-PCR	*gyrA*, *parC*, *qnrA, qnrB, qnrS, aac(6′)-Ib*	[96]
Benmahmod et al., 2019	50	-	98	AST by KB, PCR, RAPD	*bla*_OXA-51_, *bla*_OXA-23_, *bla*_OXA-58_, *bla*_OXA-24_, *bla*_SIM_, *bla*_NDM_, *bla*_VIM_, *bla*_IMP_, *bla*_KPC_, *bla*_GES_	[97]
Al-Hassan et al., 2019	59	-	93	Vitek 2, MLST	*bla*_OXA-51_, *bla*_OXA-23_, *bla*_OXA-58_, *bla*_NDM-1_*bla*_VIM-1_	[98]
Abouelfetouh et al., 2019	74	-	100	AST by KB, PCR	*bla*_OXA-51_, *bla*_OXA-23_, *bla*_OXA-58_, *bla*_NDM*,*_*bla*_VIM_	[99]
Attia and Elbaradei, 2019	21	76	100	MALDI-TOF-MS, PCR	*bla*_OXA-51_, *gyrA*, *parC*	[100]
El-Far et al., 2019	160	73	89	Vitek 2, POT	-	[101]
Tolba et al., 2019	45	100	89	PCR, Vitek 2	*bla*_OXA-51-like_, *bla*_OXA-23-like_, *bla*_OXA-24-like_, *bla*_OXA-58-like_	[102]
Ghanema et al., 2019	7	100	86	Vitek 2	-	[104]
Elbrolosy et al., 2019	64	100	84	Vitek 2, MHT, CDT, PCR	*bla*_NDM-1_	[103]
Wassef et al., 2020	12	-	-	Chromagar, Vitek 2	-	[105]
Elsayed et al., 2020	30	100	84	AST by KB, Vitek 2	-	[106]
Farag et al., 2020	6	100	83	Chromagar, Vitek 2	-	[107]
Al-Hassan and Al-Madboly, 2020	54	100	81	API 20 NE, MALDI-TOF-MS, PCR, MLST	*bla*_OXA-23_, *bla*_NDM_,*_,_ bla*_VIM-2_	[108]
Mabrouk et al., 2020	129	95.3	98	AST by KB, mCIM, CDT, BCT, PCR	*bla*_IMP_, *bla*_KPC_, *bla*_NDM_, *bla*_OXA-48_, *bla*_VIM_	[109]
El-Kazzaz et al., 2020	23	100	50	API 20NE, PCR, DNA sequencing, MHT, RAPD-PCR	*bla*_OXA-23_, *bla*_OXA-24_, *bla*_OXA-51_, *bla*_OXA-58_, *bla*_IMP_, *bla*_KPC_, *bla*_NDM_, *bla*_GES_, *bla*_VIM_	[110]
Ramadan et al., 2020	7	100	71.4	VITEK 2, PCR	*bla*_NDM_, *bla*_TEM_, *bla*_CTX-M_	[111]
Makharita et al., 2020	39	82	48.7	AST by KB, PCR, MHT, CDT, DNA sequencing	*bla*_KPC_, *bla*_GES_	[112]
Fam et al., 2020	17	-	100	Vitek 2, PCR, WGS	*bla*_OXA-51_, *bla*_OXA-23_, *bla*_NDM_, *bla*_GES_	[113]
Fam et al., 2020	22	-	100	API 20NE, Vitek 2, AST by KB	-	[114]
Khodier et al., 2020	48	-	63	Vitek 2, PCR	*bla*_OXA-51-like_, *bla*_OXA-23-like_, *bla*_OXA-24-like_, *bla*_OXA-58-like_, class 1 integrons	[115]
Abd El-Baky et al., 2020	20	60	20	PCR, DDST, MHT	*bla*_CTXM-15_, *bla*_Oxa-51,_ *bla*_Oxa-23_, *bla*_Oxa-143_	[116]
M Shabban et al., 2020	14	100	85	Real-time PCR, Etest	*mcr-1*	[117]
ELsheredy et al., 2021	100	-	-	Vitek 2, AST by KB, PCR	*bla*_OXA-51_, *aphA6*, *aphA1*, *armA*	[118]
Kishk et al., 2021	52	75	36	Vitek 2, PCR	*aacC1*, *aphA6*, *addA1*	[119]
Asaad et a, 2021	94	100	75	API 20NE, Vitek 2, Etest, PCR	*bap*, *ompA*, *bla*_PER-1_	[120]
Wasfi et al., 2021	48	-	70.8	CHROMagar, MALDI-TOF-MS, Vitek 2, PCR, ERIC-PCR, MLST	*bla*_OXA-51_, *bla*_OXA-23_, *bla*_NDM_, *bla*_KPC_, *bla*_GIM_, *bla*_SPM_, *bla*_SIM_, *bla*_IMP_, *bla*_OXA-58_, *bla*_OXA-23/24_	[121]
Hassan et al., 2021	206	-	100	PCR, AST by KB, PCR, DNA sequencing	*bla*_OXA-51_, *bla*_OXA-23_, *bla*_OXA-58_, *bla*_NDM-1_, *bla*_SPM_, *bla*_VIM_, *bla*_SIM-1_, *bla*_KPC_	[122]
Meawed et al., 2021	54	-	-	Vitek 2, AST by KB	-	[123]
Mohammed et al., 2021	100	100	-	PCR, AST by KB, DNA sequencing, ERIC-PCR	*gyrA*, *parC*	[124]
Khalil et al., 2021	54	-	97	API 20NE, Vitek 2, AST by KB, THT, PCR,	*bla*_OXA-23_, *bla*_NDM_, *bla*_PER-1,_ *bap*	[125]
Zafer et al., 2021	20	100	100	Vitek 2, PCR, AST by KB, MLST, WGS	*bla*_NDM_, *bla*_VIM_, *bla*_IMP_	[126]
Jalal et al., 2021	31	100	100	Vitek 2, WGS	-	[127]
Saleh and El-Sayed, 2021	52	98	100	Vitek 2, MHT, CDDT, PCR	*bla*_IMP-1_	[130]
Abouelfetouh, 2022	54	-	100	Vitek 2, MALDI-TOF-MS, WGS	*bla*_OXA-23_, *bla*_NDM-1_, *bla*_NDM-2_	[128]
Hamed et al., 2022	20	100	100	Vitek 2, AST by KB, PCR, WGS	*bla*_OXA-51_	[129]

Abbreviations: Antibiotic susceptibility testing (AST), Combined Disk Test (CDT), Blue-Carba Test (BCT), Carba NP test (CNPt), double-disc synergy test (DDST), Enterobacterial repetitive intergenic consensus (ERIC), isoelectric focusing (IEF), inhibitor-potentiated disk diffusion (IPD), Kirby Bauer (KB), Matrix-assisted laser desorption/ionization-time of flight mass spectrometry (MALDI-TOF-MS), Modified Carbapenem Inactivation Method (mCIM), Modified Hodge Test (MHT), Multilocus sequence typing (MLST), Polymerase chain reaction-based open reading frame typing (POT), Pulsed-field gel electrophoresis (PFGE), Triton Hodge Test (THT), Pulsed-field gel electrophoresis (PFGE), Random amplified polymorphic DNA (RAPD), Restriction Fragment Length Polymorphism (RFLP), Whole genome sequencing (WGS).

**Table 3:** Natural products showed activity against *A. baumannii* by researchers from the UK and Egypt.

Study	Natural Product (NP)	Specifications of NP	Observed Activity	Ref.
**United Kingdom:**				
Betts and Wareham, 2014	Curcumin andand EGCG	Pure compounds with purity over 90% were commercially purchased	Enhanced antibacterial activity	[131]
Betts et al., 2016	Curcumin	Curcumin >95% purity was commercially purchased	Synergism with polymyxin B	[132]
Halstead et al., 2017	Engineered honey SHRO	SurgihoneyRO (SHRO) (Matoke Holdings, UK) is a licensed CE marked sterile topical engineered honey. It exerts its effect through producing higher ROS such as H_2_O_2_.	Biofilm detachment	[133]
Betts et al., 2017	Theaflavin and epicatechin	Epicatechin >90% and theaflavin >95% purity, were commercially purchased	Enhanced antibacterial activity	[134]
**Egypt:**				
Abd El-Malek et al., 2017	Manuka honey	Manuka honey obtained from Australian company, was dissolved in acidulated water, pH = 2. Fractionated using liquid chromatography and the diethyl ether fraction was rich in phenolics such as isoferulic acid, luteolin, andand chrysin	Antibacterial activity	[135]
Ibrahim et al., 2017	Aniseeds waste residue and Star anise waste residue extracts	Anise and star anise fruits were hydrodistilled to obtain the VOs. Also, the post-distillation remaining water was freeze dried and tested	Synergistic activity with antibiotics	[136]
Salem et al., 2018	*Schinus terebinthifolius*	VOs were obtained by hydro distillation and its components were identified using GCMS. Other extracts were extracted with acetone and *n*-hexane, separately, and their components were identified by GCMS and spectroscopic determination of phenolics.	Antibacterial activity	[137]
Ahmed et al., 2018	Aerial part of some medicinal plants from family Lamiaceae	Essential oils from the shoots of all plants were obtained by hydro distillation. The composition of the oils was identified using GCMS.	Antimicrobial activity	[139]
Salem et al., 2018	Essential oils	Essential oils of 10 plants commonly used in Egypt were purchased. The characterization of oil was not discussed	Antibacterial activity	[138]
Elamary et al., 2020	*Acacia nilotica*	Aqueous extract was obtained by macerating the plant into hot distilled water and filtered on 0.45 µm disk filters and its components were identified by GCMS.	Antibacterial activity	[140]
Ismail et al., 2020	*Pimenta dioica and racemosa*	Leaves and berries of *Pimenta dioica,* were separately hydro distilled to obtain VO. The VO composition was assessed by GCMS.	Antibacterial and antibiofilm activities	[141]
Gaber et al., 2020	Bee products	Honey was obtained from hives and was diluted as 50% *w*/*v* solution in Muller-Hinton broth. Propolis was gathered by collecting the hive scrapings and placed in water and heated in oven for 2 h. Wax was collected from surface, while propolis was at the bottom of container. Bee venom was collected using electric shock method.	Antibacterial activity	[144]
Salem et al., 2021	Star anise	Star anise powder was extracted with methyl *ter*-butyl ether: water 3:1 *v*/*v* followed by methanol: water 3:1 *v*/*v* and the aqueous methanol extract was used for the study, and its components were identified by LCMS	Antibacterial and antibiofilm activities	[142]
El-Shiekh et al., 2021	*Caralluma quadrangula*	The plant was extracted with methanol and fractionated with methylene chloride and *n*-butanol. From the *n*-butanol fraction russeliosides A-D, pregnane glycosides, were isolated. In addition to a flavonoid glycoside Rus. E	Antibacterial and antibiofilm activities	[143]
Mahmoud et al., 2021	*Syzygium aromaticum*	Seeds were extracted, separately, with hot water, ethanol and ethyl acetate and were analyzed using GCMS	Antibacterial activity	[146]
Sherif et al., 2021 and Abdelaziz et al., 2021	Cinnamic and gallic acids	Cinnamic and gallic acid were purchased commercially	Antibacterial and antibiofilm activities	[147,148]
Abd El-Wahed et al., 2021	beehive air volatiles	Beehive air is a representative sample consisting of propolis: honey: wax: bee bread: royal jelly: larvae drones: larvae queen: venom (10:10:10:10:10:10:1:1:1:0.2), respectively. The constituents were analyzed by solid phase microextraction SPME-GCMS	Antibacterial activity	[145]
Amer et al., 2021	*Nigella sativa* and *Lawsonia inermis*	The powdered plants were extracted with ethanol using Soxhlet apparatus.	Antibacterial activity	[149]

## Data Availability

Not applicable.
